# Dr. Edward H. Ballou

**Published:** 1908-08

**Authors:** 


Dr. Edward H. Ballou, of Gardenville, N. Y., died at his home in that village July 25, 1908, aged fifty-four years. He received his preliminary education at the Clarence Academy and graduated in medicine at the University of Buffalo in 1878. He almost immediately located at Gardenville and for twenty years served as health officer of the town of West Seneca. He was one of the founders of Gardenville high school and was president of the board of trustees from the beginning. He was a member of the Medical Society of the County of Erie, of which he was president a few years ago. Dr. Ballou is survived by his wife, (nee Bertha Hafner), two daughters, Bertha L. Ballou and Mrs. Alfred Frey, of Buffalo, and one son, Edward J. Ballou.

